# Efficient Optical Modulation of Exciton State Population in Monolayer MoS_2_ at Room Temperature

**DOI:** 10.3390/nano12183133

**Published:** 2022-09-09

**Authors:** Zeqian Ren, Qiwei Zhang, Xiu Li, Lixia Guo, Jizhou Wu, Yuqing Li, Wenliang Liu, Peng Li, Yongming Fu, Jie Ma

**Affiliations:** 1 State Key Laboratory of Quantum Optics and Quantum Optics Devices, School of Physics and Electronic Engineering, Institute of Laser Spectroscopy, Shanxi University, Taiyuan 030006, China; 2 Collaborative Innovation Center of Extreme Optics, Shanxi University, Taiyuan 030006, China

**Keywords:** transition metal dichalcogenides, monolayer MoS_2_, photoluminescence, optical modulation, exciton state population, exciton-exciton annihilation

## Abstract

The modulation of exciton energy and state density of layer-structured transition metal dichalcogenides (TMDs) is required for diverse optoelectronic device applications. Here, the spontaneous inversion of exciton state population in monolayer MoS_2_ is observed by turning the pump light power. The excitons prefer to exist in low energy state under low pump power, but reverse under high pump power. To discuss the mechanism in depth, we propose a semiclassical model by combining the rate equation and photo−exciton interaction. Considering the modifying of exciton−exciton annihilation, the spontaneous inversion of exciton state population is phenomenologically described.

## 1. Introduction

Transition metal dichalcogenides (TMDs) have emerged as promising semiconductors that provide powerful tools for studying light−matter interaction, such as valley electronics, spin electronics, nano photonics, and nonlinear optics [1,2,3,4,5,6,7,8,9]. The bulk TMDs with structural formula MX_2_ (M = Mo and W, X = S, Se, and Te) have been properly investigated as narrow-bandgap semiconductors (0.8–1.3 eV) with layered structure and hexagonal symmetry [10,11,12,13,14]. Once the bulk TMDs are thinned to 2H-phase monolayers, the bandgaps transform from indirect to direct in the range of 1.6–2.1 eV [15,16,17]. Simultaneously, a minor energy splitting in conduction band and a major energy splitting in valence band are resulted with opposite spins in the 2H-phase monolayers [16]. As a result, the reduced dimensionality and highly nonlocal dielectric screening of Coulomb interaction give rise to large exciton binding energies (0.1–0.6 eV), leading to the richly excitonic photoluminescence (PL) and differential reflection spectra [18,19,20,21,22,23]. Therefore, the modulation of exciton state plays an essential role in the studies of monolayer TMDs.

Typically, monolayer MoS_2_ is a good room-temperature exciton material with high exciton binding energy (~150 meV). Due to the strong spin−orbit coupling (SOC) and inversion symmetry breaking, the exciton energy level splits into two energy levels with opposite spins and different energies [24]. The excitons with lower and higher energies are labeled as A-exciton and B-exciton, respectively, which dominate the optical response and quantum electrodynamics [25]. Although many approaches have been done to modulate the exciton property of monolayer MoS_2_, it is confusing that the energy and population of A-exciton and B-exciton are generally unrepeatable in diverse previous reports. Some researchers concluded that the excitons are easily modulated by environmental conditions, such as electric field, magnetic field, temperature, strain, and substrate [26,27,28,29,30,31,32,33,34]. However, the spontaneous conversion relating to pump light has not been systematically studied yet.

In this work, the relationship between the exciton state and pump light in monolayer MoS_2_ is experimentally studied using PL spectrum. In a PL spectrum, the positions and intensities of the two peaks serve as the energy and state density of A-exciton and B-exciton, respectively. Interestingly, it is found that the intensity ratio of B-exciton and A-exciton does not display a monotonous increase or decrease with the increasing power of pump light. Under pump light in low-power range, the intensity ratio decreases with increasing power, while reverses under high-power range. To discuss the mechanism, we have developed a novel theory by combining the rate equations describing the dipole transitions and the semi-classical theory describing the interaction between electron and photon, which can be concluded that the exciton population states in monolayer MoS_2_ significantly depend on the pump power in presence of exciton−exciton annihilation (EEA).

## 2. Experimental

### 2.1. Synthesis of Monolayer MoS_2_

Monolayer MoS_2_ was synthesized on a SiO_2_/Si substrate through chemical vapor deposition (CVD) method in a tube furnace (TZJ−1200, Tianjin Zhonghuan (Tianjin, China)). 0.01 g of MoO_3_ powder in a combustion boat covered with a piece of SiO_2_/Si substrate was held at the middle of tube furnace, and 0.5 g of S powder was placed 15 cm away from the MoO_3_ powder in front. Before heating, the tube was vacuumized and filled with pure N_2_, repeated three times. Then, the tube was heated to 810 °C with a heating rate of 15 °C min^−1^ under 70 sccm of N_2_ flow. After heating for 30 min, the tube was naturally cooled down to room temperature and the monolayer MoS_2_ was successfully synthesized on the lower surface of the SiO_2_/Si substrate.

### 2.2. Characterization and Measurements

The morphology of monolayer MoS_2_ was observed by an optical microscope and scanning electron microscope (SEM, SU−8010, Hitachi (Tokyo, Japan)). Raman and PL spectra were obtained by a homemade laser scanning confocal microscope. The pump light was provided by a CW laser (532 nm, 50 mW) and the spectra were acquired by a highly sensitive grating spectrometer (Nemo-N532-Spec01, Beijing Zeyou (Beijing, China)) with an integral time of 100 ms. The laser was focused into a light spot with a diameter of ~1 μm by a plan semi-apochromat objective lens (MPLFLN100X, OLYMPUS (Tokyo, Japan)) to irradiate the monolayer MoS_2_ held on a 3D-motorized positioning stage for spectrum measurements. During the low-power test, the pump light was attenuated to ~10^−4^ order of the initial power by passing through a neutral density filter (NE530B, Thorlabs (Newton, NJ, USA)), and the integral time was extended to ~30 s to improve the signal-to-noise ratio of PL spectra.

## 3. Results and Discussion

### 3.1. Characterizations of Monolayer MoS_2_

**Figure 1:**
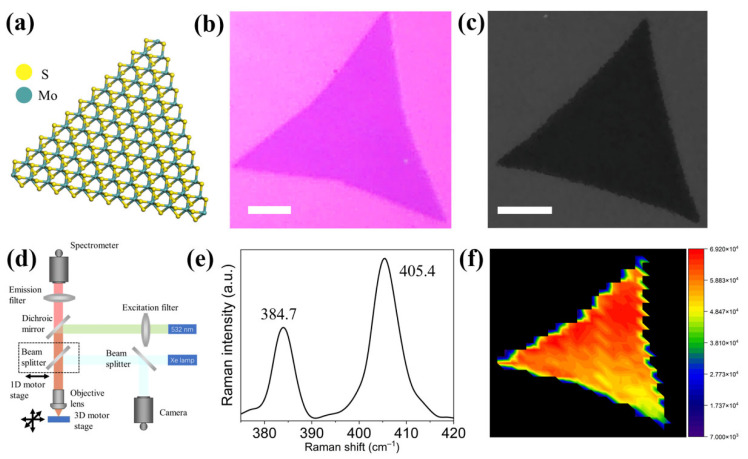
(**a**) Schematic illustration of the atomic structure of a monolayer MoS_2_. (**b**) Optical photograph of a monolayer MoS_2_. (**c**) SEM image of a monolayer MoS_2_. Scalar bar is 10 μm. (**d**) Structure of the homemade laser scanning confocal microscope. (**e**) Raman spectrum of the monolayer MoS_2_. (**f**) Raman mapping of the monolayer MoS_2_.

The atomic structure of CVD-synthesized 2H-phase monolayer MoS_2_ is schematically illustrated in Figure 1a, showing an asymmetry in the horizontal direction. To characterize the morphology of the monolayer MoS_2_, an equilateral triangle with a smooth side length of ~30 μm can be observed by an optical photograph (Figure 1b), which is in good consistent with the SEM image (Figure 1c). To identify the uniform thickness and optical property of the MoS_2_, a homemade laser scanning confocal microscope is utilized to study the Raman and PL spectra (Figure 1d). Figure 1e shows the room-temperature Raman spectrum of the MoS_2_. The two peaks located at 384.7 and 405.4 cm^−1^ correspond to $E_{2g}^{1}$ in-plane mode and $A_{1g}$ out-plane mode of MoS_2_ crystal, respectively [35]. The peak distance of 20.7 cm^−1^ agrees well with the character of monolayer MoS_2_. The universal monolayer region of the MoS_2_ crystal can also be directly determined by the means of Raman mapping calculated by the intensity of $A_{1g}$ peak (Figure 1f).

**Figure 2:**
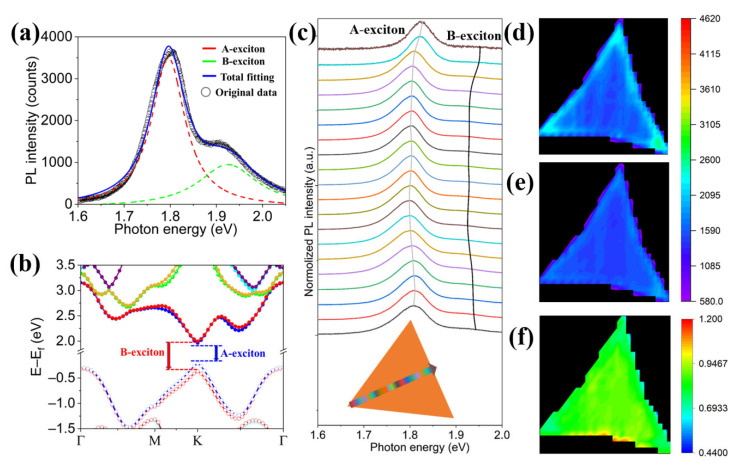
(**a**) A typical PL spectrum of the monolayer MoS_2_. (**b**) The SOC-dependent band structure of the monolayer MoS_2_. The solid and hollow circle represent conduction and valence band, respectively. The energy levels are marked by different colors. (**c**) The PL spectra with linear scanning from the vertice to edge of the MoS_2_ triangle. The inset shows the scanning path and the colors of PL spectra indicate the site position. (**d**–**f**) The intensity mapping profiles of (**d**) A-exciton, (**e**) B-exciton, and (**f**) intensity ratio of B/A.

Figure 2a presents a typical PL spectrum of the monolayer MoS_2_ excited by 532 nm laser at room temperature. The PL spectrum can be perfectly divided into two peaks by the Lorentz formula with R^2^ = 0.99. The major peak at 1.83 eV and the minor peak at 2.02 eV are raised from the dipole transitions of A-exciton and B-exciton, respectively [35]. To better understand the fundamental formation of the excitons, the electronic structure of monolayer MoS_2_ is calculated by density functional theory (DFT) with the pseudopotential plane-wave method, as shown in Figure 2b. It is intuitively found that the valence band maximum (VBM) and conduction band minimum (CBM) of the monolayer MoS_2_ are located at K-point, demonstrating a direct bandgap character. Driven by the strong SOC, both the VBM and CBM split into two bands with opposite spins. The VBM splitting is ~300 meV, which is attributed to the high atomic number of Mo atoms [36,37,38]. The CBM splitting is merely ~15 meV, which is ignored for simplification. Once the monolayer MoS_2_ is irradiated with a 532 nm laser, the electrons in VBM are excited to CBM and the holes are left in VBM. The electrons in CBM and the holes in VBM with the same spin are attracted by each other to form two bound states, named “exciton”. Generally, the bonded electron−hole pairs with lower and higher energies are called A-exciton and B-exciton, respectively. According to the previous report, the PL properties of monolayer TMDs are dominated by exciton recombination [39]. Figure 2c shows the position-dependent PL spectra of the monolayer MoS_2_ along the vertice-to-edge direction at 1 μm interval, indicating a red-shift of exciton peaks orienting from center to edge. Accordingly, the center of MoS_2_ exhibits the maximum intensities for both A-exciton and B-exciton. To further study the excitonic population in MoS_2_, the PL intensity mapping profiles of A-exciton and B-exciton are shown in Figure 2d,e, respectively. The intensities of A-exciton and B-exciton are both highest at the center area and gradually decrease toward all directions, which is caused by the fringe effect. Figure 2f shows the peak intensity ratio of B-exciton to A-exciton, depicting that the proportion is almost constant throughout the triangle. These results indicate that although the density and energy of exciton state are non-constant under the same pump light, the exciton population is constant. Therefore, to conduct the comparison experiments, the PL spectra must be obtained from the same region of the monolayer MoS_2_. In this case, the following experiments are all excited from the center of MoS_2_ triangle.

### 3.2. Pump Power Dependent PL Properties

**Figure 3:**
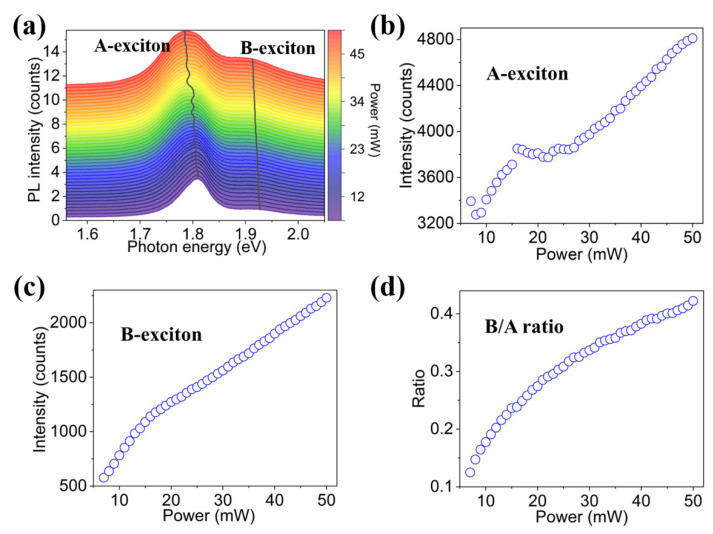
(**a**) The PL spectra of the monolayer MoS_2_ pumped by laser with high power ranging from 5 to 50 mW. (**b**–**d**) The power-dependent (**b**) intensity of A-exciton, (**c**) intensity of B-exciton, and (**d**) intensity ratio of B/A.

To quantify the population of exciton states in excited monolayer MoS_2_, the room-temperature PL spectra of the monolayer MoS_2_ are measured under 532 nm laser with high power evenly ranging from 5 to 50 mW, as shown in Figure 3a. All the PL spectra can be well fitted by the linear superposition of the emission peaks of A-exciton and B-exciton. It can be observed that the energies of A-exciton and B-exciton slightly red-shift with increasing power, which is ascribed to the laser-induced photothermal effect [40]. The peak intensities of A-exciton and B-exciton under different pump powers are shown in Figure 3b,c, respectively, showing a linearly positive correlation between exciton state density and pump power. Figure 3d displays the intensity ratio of B-exciton to A-exciton, which indicates that the B/A intensity ratio is near-linearly and positively related to the pump power. These results suggest that the low-energy A-exciton can spontaneously convert to high-energy B-exciton with increasing pump power in the relatively high region.

**Figure 4:**
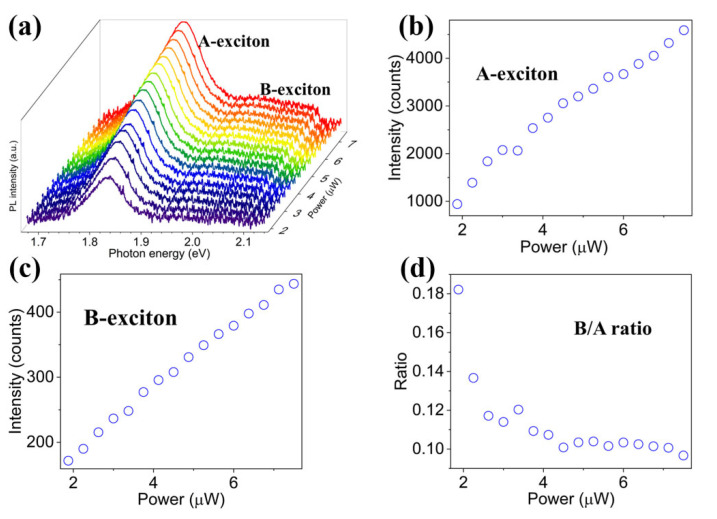
(**a**) The PL spectra of the monolayer MoS_2_ under pump light with low power ranging from 1.875 to 7.85 μW. (**b**,**c**) The intensity variations of (**b**) A-exciton and (**c**) B-exciton under increasing pump power. (**d**) The ratio variation of B/A intensity ratio under increasing pump power.

To investigate the variation tendency of exciton state population under low-power pump light, the room-temperature PL spectra of the monolayer MoS_2_ under 532 nm laser with pump power ranging from 1.875 to 7.85 μW are measured, as shown in Figure 4a. Although the peak intensities around 2.02 eV are much weaker than those around 1.83 eV, all the PL spectra can still be well fitted by two Lorentzian curves assigned to B-exciton and A-exciton located at constant positions. Moreover, both the two peak intensities of A-exciton and B-exciton increase with the increasing pump power (Figure 4b,c). Astonishingly, different from the PL spectra under high pump power, here the intensity ratio of B-exciton to A-exciton nonlinearly decreases while the pump power increases (Figure 4d). Therefore, it can be concluded that the pump light with low power prefers to generate A-exciton but reverses under high power, which has not yet been reported in the previous literature.

### 3.3. Proposed Mechanism

**Figure 5:**
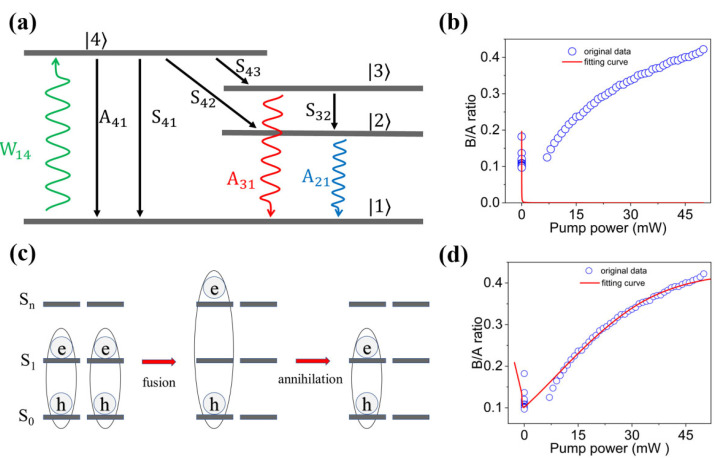
(**a**) Schematic diagram of the exciton state population of the monolayer MoS_2_ during excitation and recombination processes. (**b**) The theoretical calculation result without EEA-modification. (**c**) Schematic diagram of exciton−exciton fusion and annihilation processes. (**d**) The theoretical calculation result with EEA-modification.

To discuss the abnormal exciton state population in monolayer MoS_2_ at room temperature, we have firstly developed a simple four-level model, as shown in Figure 5a.

When the monolayer MoS_2_ is irradiated by a 532 nm laser, the electrons in ground state |1〉 are pumped to high-energy excited state |4〉. Then, the metastable A-excitons and B-excitons originated from non-radiation intra-band relaxation and electron−hole Coulomb interaction are generated in state |2〉 and |3〉, respectively. Subsequently, the excitons in state |2〉 and |3〉 relax to state |1〉 through spontaneous radiation, forming the related peaks in PL spectra. Likewise, the electrons in the state |4〉 relax to the state |1〉 through spontaneous radiation and non-radiation transition. For simplify, the detailed electron−hole interaction and the non-radiation transition with low possibility are neglected. According to the Purcell effect, the spontaneous emission rate from state |4〉 to |1〉 depends on the pump power [41,42]. The population of the four quantum states can be calculated from the phenomenological formula, as described by Equations (1)–(4) [43],

(1) $$\frac{dn_{4}}{dt}=n_{1}W_{14}-n_{4}\left(A_{41}+S_{41}+S_{43}+S_{42}\right)$$

(2) $$\frac{dn_{3}}{dt}=n_{4}S_{43}-n_{3}\left(A_{31}+S_{31}+S_{32}+A_{41}+S_{41}\right)$$

(3) $$\frac{dn_{2}}{dt}=n_{4}S_{42}+n_{3}S_{32}-n_{2}\left(S_{21}+A_{21}\right)$$

(4) $$n_{1}+n_{2}+n_{3}+n_{4}=n$$

where *n* represents the total state density, *n_j_* represents the state density of excitons and electrons in energy level *E_j_*, *S_ij_*, *A_ij_*, and *W_ij_* represent the non-radiation transition rate, spontaneous emission rate, and stimulated absorption rate from level |i〉 to |j〉, respectively. All the computations and analyses are based on the steady-state assumption that the state densities in the four levels are constant in the whole process, as expressed by $\frac{dn_{i}}{dt}=0$. Therefore, the exciton state population can be described by Equation (5),

(5) $$\frac{I_{B}}{I_{A}}=\frac{A_{31}S_{43}}{\left(A_{31}+A_{41}+S_{32}\right)S_{42}+S_{32}S_{43}}$$

where *I_B_* and *I_A_* represent the PL intensities corresponding to *A*_31_ and *A*_21_, respectively.

Furthermore, a semi-classical model is developed to analyze the dependence of spontaneous emission rate on the pump power by discussing the light−matter interaction. The single-mode electromagnetic wave is expressed in classical form by Equation (6) [44],

(6) $$E\left(t\right)=E_{0}cos\left(\omega t\right)=\frac{1}{2}E_{0}\left(e^{i\omega t}+e^{-i\omega t}\right)$$

where ***E***_0_ and *ω* are the amplitude and angular frequency of incident electromagnetic wave, respectively. By solving a time-dependent Schrödinger equation with perturbation method and diploe approximation, the light−matter interaction can be written as Equations (7) and (8) [44],

(7) $$i\hbar \frac{d}{dt}|\psi \left(t\right)\rangle =H|\psi \left(t\right)\rangle$$

(8) $$H=H_{A}+V$$

where *V* and *H_A_* correspond to the interaction Hamiltonian representing the perturbation part and the free particles Hamiltonian, respectively. Since the scale of atom is much smaller than that of photon, the interaction part can be expressed as Equation (9) through dipole approximation [45],

(9) V=−d⋅E

where ***d*** is the electric dipole moment, expanded with the eigenstate of *H_A_* by Equation (10).

(10) $$d=\underset{j,k}{\sum }d_{jk}|j\rangle \langle k|$$

Thus, the transition probability *Ƥ_ni_(t)* can be calculated through perturbation method and rotating approximation, as described by Equation (11),

(11) $$P_{ni}\left(t\right)={\left|\frac{E_{0}\cdot d_{ni}}{\hbar^{2}}\right|}^{2}{\left|\frac{\sin\left[\left(\omega_{ni}-\omega \right)t/2\right]}{\left(\omega_{\mathrm{ni}}-\omega \right)}\right|}^{2}$$

where *ω_ni_* is the resonant angular frequency between state |n〉 and |i〉 and *Ƥ_ni_(t)* is of positive correlation with pump power (as well as |***E***_0_|^2^). As the difference between *ω_ni_* and *ω* is of non-zero value, *Ƥ_ni_(t)* dramatically approaches to zero with calculating the average value. By combining Equations (2) and (8), the B/A intensity ratio has a negative correlation with pump power, as fitted in Figure 5b. The fitting curve displays that the B/A ratio rapidly decreases with increasing pump power and then trends to be gentle. Obviously, the simple four-energy-level model can well describe the variation of exciton state population in low-power region, but is not applicable in high power region, where the mismatch may be attributed to the EEA under high power laser irradiation since the EEA can only be obtained under high photon density.

**Figure 6:**
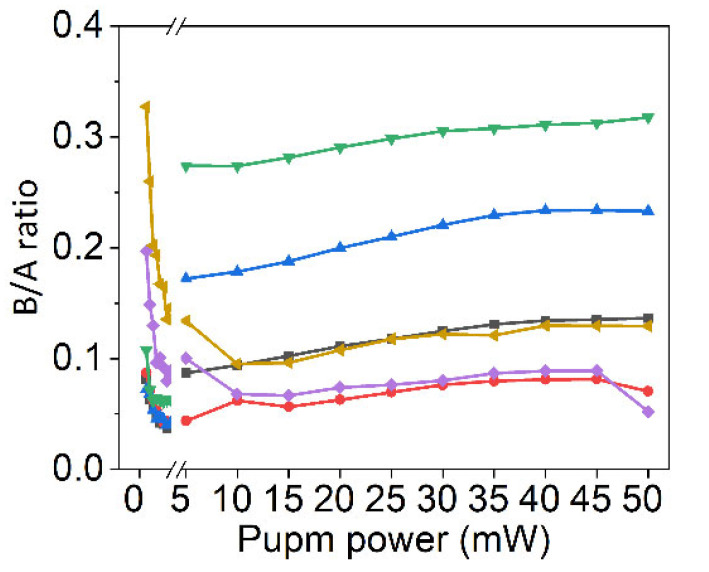
The variation of the B/A intensity ratio of 6 selected sites from the same sample after keeping in air for 6 months. The B/A ratios raised form different sites are labeled by different colors.

Subsequently, we have further developed an EEA-modified four-energy-level model to improve the accuracy. For simplifying the calculation, the energy level structure of exciton state is regarded as a three-energy-level model including ground state *S_0_*, first excited state *S_1_*, and high-order excited state *S_n_* by neglecting other states due to the large detuning between resonance frequency and photon angular frequency, as shown in Figure 5c. In the EEA model, both the state densities of A-exciton and B-exciton existing in state *S_1_* increase with increasing pump power. As the pump power increases to the threshold value, EEA occurs between two nearby A-excitons because the state density of A-exciton is much higher than that of B-exciton. One A-exciton is pumped to high-order excited state with double energy of A-exciton level through energy transfer in exciton fusion process, and another A-exciton is recombined to ground state through non-radiation route. The exciton density *n_x_(**r**,t)* containing annihilation term is proportional to the square of state density, as described by Equation (12) [46],

(12) $$\frac{\partial n_{x}\left(r,t\right)}{\partial t}=G\left(r,t\right)+D\nabla^{2}n_{x}\left(r,t\right)-\frac{n_{x}\left(r,t\right)}{\tau_{x}}-C_{EEA}n_{x}^{2}\left(r,t\right)$$

where ***r***, *t*, *G*, *D*, *τ_x_*, and *C_EEA_* represent the position, time, particle generation rate, exciton diffusion coefficient, single exciton lifetime, and EEA coefficient, respectively. In the case of single point excitation and steady states, there is one more term *C_EEA_n_x_*^2^ to describe the EEA process than that under low exciton density. The phonon emission induced out-band conversion results in a rapid relaxation, leaving only one exciton after EEA process. Therefore, by considering the EEA process, Equations (2) and (3) can be improved to Equations (13) and (14) [46],

(13) $$\frac{dn_{3}}{dt}=n_{4}S_{43}-n_{3}\left(A_{31}+S_{31}+S_{32}+A_{41}+S_{41}\right)-C_{3}n_{3}^{2}$$

(14) $$\frac{dn_{2}}{dt}=n_{4}S_{42}+n_{3}S_{32}-n_{2}\left(S_{21}+A_{21}\right)-C_{2}n_{2}^{2}$$

where *C*_2_ and *C*_3_ correspond to the EEA coefficient in level |2〉 and |3〉, respectively. Since the EEA-modified equations of exciton state population are so complicated that could not be expressed in simplified form, Equation (5) is still used to calculate the exciton state population by ignoring EEA rate. The numerical solution is re-calculated through assigning the certain radiation rate and non-radiation rate obtained from other works, as shown in Figure 5d. Typically, the values of *S*_43_ (*S*_42_), *S*_32_, and *A*_31_ are approximated as ~60 fs, 1 ps, and 60 ps, respectively [47,48]. The radiation rate of *A*_41_ is a variate related to the total exciton number, which dominates the non-linear exciton population under varying pump power. With increasing pump power, the calculated curve no longer monotonically decreases, and a valley appears. Excitingly, the EEA-modified fitting curve exhibits a high coincidence with experimental data in both lower-power and higher-power region. These results suggest that EEA plays an important role in the spontaneous inversion of exciton states in monolayer MoS_2_. Excitons are quasi-particles, a kind of Boson. Under low pump power, the exciton number is relatively low, leading to the normal Bose-Einstein distribution of excitons. Since the energy level of A-exciton is lower than B-exciton, the excitons in A-state are more than B-state. Under high pump power, the exciton number is relatively high, and EEA must be considered. With the increasing pump power, the portion of B-excitons increases correspondingly, and the decay of EEA in A-excitons is faster than B-excitons [49,50]. Therefore, it can be concluded that the excitons prefer to exist in a low energy state under low pump power while reverses under high pump power. In addition, the PL spectra of the same sample are measured after keeping in air for 6 months to study the effect of defects since the defect density increases with the increasing time in air through the oxidation of oxygen [48,51,52]. Figure 6 shows the B/A intensity ratio dependent on pump power of 6 sites randomly selected from the same sample. Although the variation tendencies are generally consistent with Figure 5d, the increasing rates in the high-power region are much lower. These results suggest that the exciton population are significantly affected by defects, which should be further studied in future works.

## 4. Conclusions

In summary, the spontaneous inversion of exciton state population in monolayer MoS_2_ at room temperature has been observed and discussed. The excitons prefer to exist in low energy state under low pump power, and reverses under high pump power. Since the non-radiation rate keeps constant in the whole excitation−recombination process, the exciton state population can be modulated by pump light through changing the transition probability of spontaneous radiation without other external factors, which is explained by the rate equation and photo-exciton interaction. By combining the relation formula from the rate equations and the final equation from semiclassical theory, the relationship between exciton state population and power of pump light is correlative to the square of transition probability of spontaneous radiation. To make up for the shortcomings of the simple four-energy-level model, EEA theory is introduced in a simplified three-energy-levels system for phenomenologically describing the spontaneous inversion of exciton state population. This work develops a novel all-optical method to modulate exciton population and discusses the EEA process in exciton transition, which provides a possible model to normalize the PL spectrum for SOC studies and contributes to further understanding the light−matter interaction in low-dimensional nanomaterials.

## Data Availability

The data presented in this study are available on request from the corresponding authors.
